# Natural Products for the Management of Castration-Resistant Prostate Cancer: Special Focus on Nanoparticles Based Studies

**DOI:** 10.3389/fcell.2021.745177

**Published:** 2021-11-05

**Authors:** Rajeev K. Singla, Chandragiri Siva Sai, Hitesh Chopra, Sahar Behzad, Himangini Bansal, Rajat Goyal, Rupesh K. Gautam, Christos Tsagkaris, Shikha Joon, Shailja Singla, Bairong Shen

**Affiliations:** ^1^Frontiers Science Center for Disease-related Molecular Network, Institutes for Systems Genetics, West China Hospital, Sichuan University, Chengdu, China; ^2^iGlobal Research and Publishing Foundation, New Delhi, India; ^3^Amity Institute of Pharmacy, Amity University, Lucknow, India; ^4^Chitkara College of Pharmacy, Chitkara University, Rajpura, India; ^5^Evidence-Based Phytotherapy and Complementary Medicine Research Center, Alborz University of Medical Sciences, Karaj, Iran; ^6^Department of Pharmacognosy, School of Pharmacy, Shahid Beheshti University of Medical Sciences, Tehran, Iran; ^7^Delhi Institute of Pharmaceutical Sciences and Research, New Delhi, India; ^8^MM School of Pharmacy, MM University, Ambala, India; ^9^Faculty of Medicine, University of Crete, Heraklion, Greece

**Keywords:** prostate cancer, advanced stage cancer, anticancer nanoformulations, site-targeted drug delivery, hormone-sensitive cancer

## Abstract

Prostate cancer is the most common type of cancer among men and the second most frequent cause of cancer-related mortality around the world. The progression of advanced prostate cancer to castration-resistant prostate cancer (CRPC) plays a major role in disease-associated morbidity and mortality, posing a significant therapeutic challenge. Resistance has been associated with the activation of androgen receptors *via* several mechanisms, including alternative dehydroepiandrosterone biosynthetic pathways, other androgen receptor activator molecules, oncogenes, and carcinogenic signaling pathways. Tumor microenvironment plays a critical role not only in the cancer progression but also in the drug resistance. Numerous natural products have shown major potential against particular or multiple resistance pathways as shown by *in vitro* and *in vivo* studies. However, their efficacy in clinical trials has been undermined by their unfavorable pharmacological properties (hydrophobic molecules, instability, low pharmacokinetic profile, poor water solubility, and high excretion rate). Nanoparticle formulations can provide a way out of the stalemate, employing targeted drug delivery, improved pharmacokinetic drug profile, and transportation of diagnostic and therapeutic agents *via* otherwise impermeable biological barriers. This review compiles the available evidence regarding the use of natural products for the management of CRPC with a focus on nanoparticle formulations. PubMed and Google Scholar search engines were used for preclinical studies, while ClinicalTrials.gov and PubMed were searched for clinical studies. The results of our study suggest the efficacy of natural compounds such as curcumin, resveratrol, apigenin, quercetin, fisetin, luteolin, kaempferol, genistein, berberine, ursolic acid, eugenol, gingerol, and ellagic acid against several mechanisms leading to castration resistance in preclinical studies, but fail to set the disease under control in clinical studies. Nanoparticle formulations of curcumin and quercetin seem to increase their potential in clinical settings. Using nanoparticles based on betulinic acid, capsaicin, sintokamide A, niphatenones A and B, as well as atraric acid seems promising but needs to be verified with preclinical and clinical studies.

## Introduction

Cancer is a major public health problem that has a significant global impact on both developed and developing countries (Bray et al., 2018; Joon et al., 2021; Qi et al., 2021). Considering the high-profile nature of the disease, its treatment has been a constant struggle with relatively less success (Choudhari et al., 2020). Prostate cancer (PCa), a hormonally driven cancer, ranks first in incidence and second in cancer-related mortality in men in most Western industrialized countries (Siegel et al., 2018; Lin et al., 2020; Chen et al., 2021). As we have long understood, prostate cancer cells demonstrate sensitivity to circulating androgens and initially respond to various methods of suppressing testosterone (Huggins et al., 1941; Ezzell et al., 2013). Despite the initial success of androgen deprivation therapy (ADT) for advanced PCa, virtually, all patients eventually develop biochemical and clinical evidence of treatment resistance. This disease status is known as castration-resistant prostate cancer (CRPC). The development of castration resistance is an inevitable pathway for the vast majority of patients with advanced prostate cancer. Recently, there have been significant breakthroughs in the understanding and management options of CRPC (Gelmann, 2002; Dent et al., 2011; Marech et al., 2012).

The World Health Organization (WHO) states that in developing countries, approximately 80% of the population relies on traditional medicine to fulfill their desired health needs (Bonifácio et al., 2013; Hussain et al., 2021). Most of the chemotherapy drugs used for treating cancers for the last 40 years have their root in natural products; furthermore, compounds from nature that are currently being characterized may provide various lead structures that can be used as templates for the synthesis of new, pharmacologically more effective agents (Mann, 2002). Natural anticancer products are found in vegetables, fruits, herbs, and fermented plant products and extracts. The anticancer activity of these products might be related to their action on cells as antioxidants, free-radical scavengers, and inhibitors of DNA-modifying enzymes. Together, these properties are likely to be protective against somatic mutations and unfavorable epigenetic DNA modifications (Reddy et al., 2003). Compounds that can prevent PCa initiation or delay its progression to CRPC should be able to reduce PCa-related mortality. Researchers have demonstrated anti-PCa activities of several natural compounds, including soy isoflavones (mostly genistein), ellagic acid and ellagitannins from pomegranate extract, green tea polyphenols, curcumin, lycopene, vitamin D, and L-selenomethionine, both *in vitro* and *in vivo* (Kallifatidis et al., 2016).

Given the challenges of collateral toxicity and non-specific distribution of PCa therapies, arising from convectional delivery methods, which translates to poor efficacy, scientists have embarked on the search for a veritable alternative to contend with these challenges.

Nanotechnology provides the platform with inherent characteristics to guarantee the safety, specificity, and therapeutic efficacy of advanced prostate cancer therapies. Nanomedicine holds the potential to improve anticancer therapy (Peer et al., 2007). Traditionally, nanomedicines are used to modulate the biodistribution and the target site accumulation of systemically administered chemotherapeutic drugs, thereby improving the balance between their efficacy and toxicity. In preclinical settings, nanomedicines typically increase tumor growth inhibition and prolong survival as compared to non-formulated drugs, but in clinical practice, patients often only benefit from nanomedicines because of reduced or altered side effects (Shi et al., 2016; van der Meel et al., 2019).

This current review will focus on natural compounds that target androgen receptor (AR)-mediated cell signaling that leads to PCa growth and progression. We will also discuss the advancement made in terms of natural product-based nanomedicines for the management of prostate cancer and advanced-stage prostate cancer.

## Pathophysiology of Castration-Resistant Prostate Cancer

### Clinical Progression of Castration-Resistant Prostate Cancer

The AR, also known as NR3C4 (nuclear receptor subfamily 3, group C, member 4), is a member of the steroid hormone group (nuclear receptors) that is activated by binding any of the androgenic hormones, including testosterone and 5α-dihydrotestosterone (DHT) in the cytoplasm and then translocating into the nucleus. The AR is most closely related to the progesterone receptor, and progestins in higher dosages can block the AR. Androgens are critical for the development and differentiation of the male sexual phenotype (Tan et al., 2015).

Testosterone, the most active androgens in males, is produced by Leydig cells in the testis. The testes are responsible for the production of 90–95% of circulating androgens, and the adrenal gland contributes the remainder. The secretion of testosterone from these cells is regulated by luteinizing hormone (LH) from the anterior pituitary gland. Serum sex hormone-binding globulin (SHBG) and albumin are responsible for transferring testosterone in the bloodstream. In target cells as well as prostate cells, 5α-reductase transforms testosterone into its active form, DHT. They can regulate the AR’s transcriptional activity. The normal function, development, and maintenance of the prostate gland are dependent on DHT acting through AR. So, androgens have an expanding role in maintaining prostate homeostasis, and under abnormal conditions, they contribute to the development of prostate cancer. Around 80–90% of cases of prostate cancers are required to measure androgen at initial diagnosis, and endocrine therapy of prostate cancer is directed toward the reduction of serum androgens and inhibition of AR (Wang et al., 2018; Kokal et al., 2020).

ADT is the mainstay of treatment against metastatic and advanced prostate cancer and is also used as an adjuvant to local treatment of high-risk diseases. Current ADT includes surgical methods such as surgical orchiectomy and medical castration including GnRH agonists and antagonists targeting the hypothalamic–pituitary axis, blocking steroid production by enzymatic inhibition, antiandrogens that inhibit binding to the AR (Komura et al., 2018).

Approximately 80–90% of patients with the metastatic disease showed quite proper clinical and biochemical responses achieving a rapid decline in serum prostate-specific antigen (PSA) at the beginning of endocrine therapy (Amaral et al., 2012) and depletion of serum testosterone to “castrate” levels (<50 ng/ml), resulting in induction of apoptosis signaling in malignant prostate cells and temporal suppression of disease (Kahn et al., 2014). However, a significant proportion of patients progress to CRPC, measured by rising serum PSA, or appearance of metastases, after an average remission time of 2–3 years. CRPC, which was previously called hormone-refractory prostate cancer, carries a poor prognosis, and it is estimated that the mean survival time of patients is 9–36 months (Čapoun et al., 2016). Approximately 90% of patients with CRPC will face bone metastases, resulting in severe pain, pathologic fractures, and/or bone marrow failure (Penticuff and Kyprianou, 2016).

The progression of tumor growth and development of metastases regardless of androgen ablation to castrate levels are caused by the utilization of several modifying cell pathways.

### Mechanisms Leading to Castration Resistance

Understanding the mechanisms underlying the progression of prostate cancer from hormone-sensitive to castration-resistant is the key to develop future therapy. Androgens and AR play a significant role in the development of CRPC. Approaching new therapeutic landscapes into effective targets will be a promising strategy in maximizing effectiveness and increasing survival in patients with CRPC (Obinata et al., 2020).

#### Backdoor Pathway for Androgens Synthesized

In adrenal and testicular cells, pregnenolone, as the immediate precursor for the synthesis of all of the steroid hormones, is raised from cholesterol and is converted to dehydroepiandrosterone (DHEA) by CYP17A enzyme in the adrenal cortex. It is also converted to testosterone *via* a series of enzymatic reactions in the testicle (Kahn et al., 2014). In the absence of gonadal androgen synthesis following ADT, the adrenal precursor DHEA is converted to androstenedione by 3β-hydroxysteroid dehydrogenase within the prostate. However, the androgen concentration in prostate tissues in castration-resistant prostates on ADT is lower than that found in normal prostates, but it is sufficient to stimulate the ARs. In addition, in patients receiving CYP17A enzyme-inhibiting drugs like abiraterone acetate, progesterone can produce DHT by steroidogenic enzymes as a backdoor pathway (Mostaghel et al., 2011; Rehman and Rosenberg, 2012; Pal et al., 2018). Studies have shown that bone metastases upregulated a group of enzymes responsible for adrenal androgen conversion to DHT or testosterone (Harris et al., 2009). Intratumoral steroidogenesis from the impaired production of cholesterol in CRPC was also observed in previous works (Leon et al., 2010; Huang et al., 2018).

#### Androgen Receptor-Dependent Mechanisms

AR exists in the luminal epithelial cells as well as stromal cells, which surround the epithelial layer. Currently, it seems AR signals remain a crucial driver of CRPC. Mutated or spliced AR, an AR bypass pathway handled by co-regulator enzymes, increased the expression of AR, and AR posttranslational modification remains the most frequently occurring AR-dependent mechanism in CRPC cases. The gene encoding AR is present in a single copy in males and is located on the X chromosome, so it allows for the phenotypic manifestation of mutations without the influence of a wild-type codominant allele (Tilki and Evans, 2014). DNA amplification with resultant overexpression of AR target gene and protein is a primary mechanism that is responsible for hypersensitivity to residual low androgen levels (Tilki et al., 2016). In several clinical studies, it is observed that over 80% of patients with CRPC showed an increment of AR mRNA and protein half-life, which may contribute to the augmentation of AR protein quantity. Moreover, the development of tumor cells constantly under ADT conditions, as well as AR overexpression, may promote CRPC formation (Haapala et al., 2007; Aggarwal et al., 2015).

Many other protein factors such as AR co-regulators are altered in hormone-resistant tumors that may activate different signal transduction pathways. More than different 150 compounds have interacted with AR as co-activators or co-repressors molecules. Insulin-like growth factor-1 (IGF1), keratinocyte growth factor (KGF), and epidermal growth factor (EGF) have been shown to enhance the activity of AR in the presence of low level or even in the absence of androgen (Watson et al., 2010).

Several novel treatment strategies provide an efficient blockade of AR signaling, and all of which have shown a significant improvement in overall survival (Narayanan, 2020).

### Castration-Resistant Prostate Cancer and the Tumor Microenvironment

The prostate tumor microenvironment is complex and heterogeneous comprising several factors, which are directly or indirectly involved in the progression of the tumor (Torrealba et al., 2017). Furthermore, this tumor microenvironment constitutes cellular entities, such as cancer-associated fibroblasts, cell infiltrates, and extracellular matrix (ECM) (Gurel et al., 2014). Fibroblasts partake in ECM formation and release collagen types I and III (Gabbiani, 2003). However, because of the inappropriate positioning of cancer, their biopsy sampling has always been a hurdle (Bjurlin and Taneja, 2014). The growth of prostatic cancer and metastasis is linked to the balance between the neoplastic cells and constituents of the stroma (Torrealba et al., 2017). The fibroblasts associated with cancer have been acting as the source of reactive stroma stimulated by PCa cells (Gabbiani, 2003; Desmoulière et al., 2005; Schauer et al., 2009; Hansen et al., 2010). The growth factors are released due to the regular destruction and formation of ECM. Also, the secretion of fibroblasts mediates the healing process. Furthermore, the release of an excessive amount of reactive oxygen species and tenascin C by cancer-associated fibroblasts promotes the proliferation and migration of prostate cancer (Schauer and Rowley, 2011). These cancer-associated fibroblasts are characterized by the presence of molecular markers, which include fibroblast activation protein, PDGFR-β, and fibroblast-specific protein-1 (Orr et al., 2011). The presence of these markers determines the heterogeneity and the origin of cancer-associated fibroblasts. The prostatic tumorigenesis is not dependent on the angiogenesis upregulation (Melegh and Oltean, 2019). Blood vessel formation plays an important role in cell viability and growth. The formation of immature leaky blood vessels characterizes the tumor vasculature. The interaction between the tumor cells and stromal endothelial cells increases the vascular endothelial growth factor (VEGF). VEGF plays an important role in the stimulation of metastatic activity and acts *via* suppression of androgen receptor and transcriptional activity. This suggests that their inhibition could stop the further replication of PCa (Amankwah et al., 2012; Tomić et al., 2012). Macrophages play an important role in tumor progression. They infiltrate into the tumor tissue and reside in the metastatic nodules (Doak et al., 2018; Loyher et al., 2018; Lin et al., 2019). The prostate-derived parathyroid hormone-related protein mediated an increase in tumor-associated macrophages (Soki et al., 2012). This parathyroid hormone-related protein recruits more myeloid cells and osteoblast-produced chemokine ligand 2 (Zhang et al., 2010). The PCa cells are also involved in the GLS1 protein expression. Due to PCa cells, the expression of GLS1 protein is enhanced compared to the benign glandular epithelium (Myint et al., 2021). A study was conducted to evaluate the importance of lysophosphatidic acid receptor 1 (LPAR1) in PCa (Shi et al., 2020). It was found that the level of LPAR1 is downregulated in patients with PCa. The role of LPAR1 can also be traced back to its involvement in the biological process; it handles the improvement of the tumor microenvironment. In the oncomine database analysis, it was observed that in the lymphoma region, the LPAR1 was over-expressed, while it was poorly expressed in the prostate, bladder, and other regions of cancer.

Inflammation has also been linked to the development of PCa, while chronic inflammation is linked to the development of malignant prostate tissue (Lu et al., 2006; Stark et al., 2015). The major region for an association of PCa and chronic inflammation is still under doubt. This may be accounted for the ongoing genotoxic stress from DNA damage that takes place during oncogenesis and may act as the major contributor (Sprung et al., 2015; Udensi and Tchounwou, 2016).

The extracellular vesicles also play an inevitable role in the paracrine communication between the cancer cells and CAFs in the prostate tumor microenvironment (Vlaeminck-Guillem, 2018). The prostate cancer cells release exosomes that harbor transforming growth factor-beta 1 (TGF-β1), which is involved in the induction of myofibroblast (MFB) transition. The disruption of PCa cells causes the loss of stroma together with their growth promotion characteristics (Webber et al., 2014). The microRNA-409, secreted by cancer-associated fibroblasts, inhibits the translation of tumor suppressor genes and helps the tumor to invade (Josson et al., 2014).

## Natural Therapeutic Products for Castration-Resistant Prostate Cancer Treatment and Management

PCa is the most commonly diagnosed cancer in males and the second most frequent cause of cancer-related mortality around the world. As stated earlier, the majority of patients with prostatic adenocarcinoma will initially respond to ADT (Zhu et al., 2013), which is taken into account because of the standard of care with locally advanced illness. However, the prolonged androgen excision is related to severe side effects, and the patients will ultimately acquire CRPC. When compared to their younger counterparts, older patients could be more prospective to develop aggressive forms of the disease (Moussa et al., 2020; Spratt et al., 2021).

The most commonly used medications for the treatment of prostate cancer are angiotensin-converting enzyme (ACE) inhibitors, β-blockers (like atenolol), statins, antiplatelet drugs, and calcium channel blockers (Saad et al., 2021). Non-steroidal AR antagonists such as flutamide, enzalutamide, and bicalutamide can selectively inhibit androgen activity with fewer adverse effects as compared to the other AR antagonists. However, after 18 months of treatment, the majority of the patients relapsed subsequently, an initial response to ADT, and acquired CRPC. Therefore, there is an urgent need to discover more efficacious AR-suppressing agents for the treatment of CRPC (Xu et al., 2019).

Research based upon natural products have been proved to be an effective methodology for discovering newer, physiologically active, and innovative medications (Cragg and Pezzuto, 2016; Singla et al., 2020a, b; Marzocco et al., 2021; Singla, 2021; Sultana et al., 2021). Several *in vitro* studies on natural products claim their excellent anticancer activity for a variety of malignancies, due to which natural products and derivatives come into focus in the field of cancer (Shen and Singla, 2020; Singla, 2020, 2021; Marzocco et al., 2021). Despite these circumstances, natural products exhibit low toxicity contours and are well endured by cancer patients (Shen and Singla, 2020; Singla, 2020; Wang et al., 2020).

### Metabolites Extracts

Rettig et al. (2008) showed that the extract of pomegranate results in the inhibition of the growth of androgen-independent PCa *via* a nuclear factor-KB-dependent mechanism. Shenouda et al. (2007) showed that an extract from the bark of an African plum tree, *Pygeum africanum*, inhibited the proliferation of PC-3 and LNCaP cells and induced apoptosis *via* downregulation of ERa and PKCa protein, which displayed a virtuous binding ability to both LNCaP human ARs and mouse uterine estrogen receptors.

### Metabolites

**FIGURE 1:**
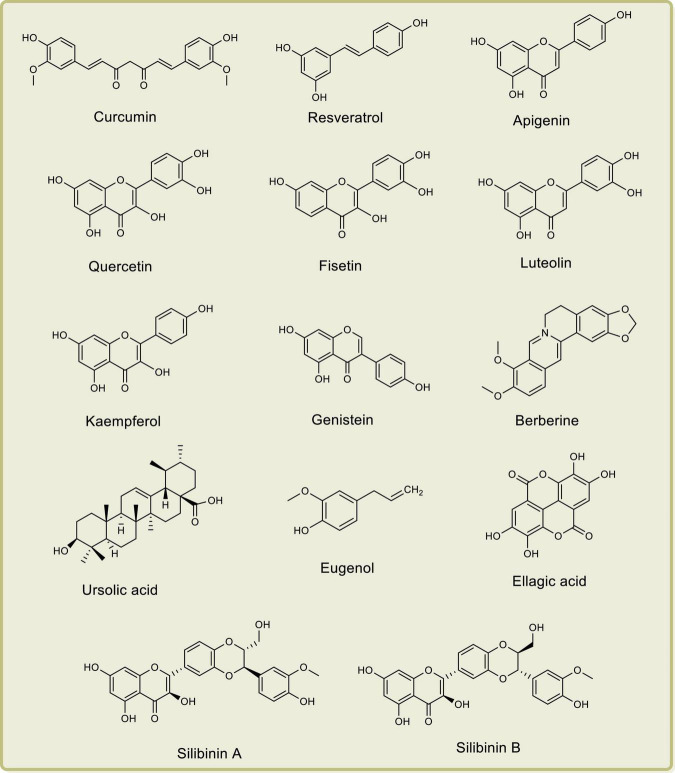
Chemical structures of important anti-prostate cancer phytochemical compounds.

Researchers have demonstrated the anti-PCa properties of several natural products such as curcumin, resveratrol, lycopene, apigenin, quercetin, fisetin, luteolin, kaempferol, genistein, berberine, ursolic acid, eugenol, gingerol, ellagic acid, silibinin A, silibinin B, and epicatechin-3-gallate, both *in vitro* and *in vivo*, by their effects on cyclooxygenases, lipoxygenases, and phospholipase A2 (Sarkar et al., 2010; Tewari et al., 2019; Fontana et al., 2020; Marzocco et al., 2021). The chemical structures of some important anti-PCa phytochemical compounds are depicted in Figure 1.

Embelin is a potential natural therapeutic agent used to prevent and treat CRPC. It demonstrated its ability to suppress numerous signaling pathways, mostly involved in the formation of cancer, and majorly *via* blocking of XIAP and caspase-9 interaction (Prabhu et al., 2018).

Curcumin is a yellow color pigment found in turmeric and derived from the plant *Curcuma longa*. It has been shown to enhance TNF and radiation-induced apoptosis in human PCa cells. It inhibits NF_k_B by the suppression of I_k_B phosphorylation, which downregulates the anti-apoptotic gene products and activates the caspases (Sainz et al., 2008). Like curcumin, pieces of evidence showed that polyphenols such as epicatechin-3-gallate (ECG) and epigallocatechin-3-gallate (EGCG) present in green tea can inhibit the methylation of DNA, which induces anticancer effects with potent anti-PCa potential (Kallifatidis et al., 2016; Wang et al., 2020). Camptothecin, a cytotoxic alkaloid, derived from the plant *Camptotheca acuminata*, exhibited noteworthy anti-proliferative activity *via* topoisomerase-I inhibition. Vinblastine, a vinca alkaloid, derived from the plant *Catharanthus roseus*, binds to tubulin and inhibits the microtubule assembly. Paclitaxel (Taxol), a mitotic inhibitor, derived from *Taxus brevifolia*, is used to treat patients with several forms of cancers (Mishra and Tiwari, 2011).

Quercetin, a penta-hydroxylated flavanol compound, found in apples, tea, onions, capers, and tomatoes, exhibited potent chemopreventive activities and cytotoxic potential by inhibiting the function of androgen receptors in LNCaP prostate cancer cells (Xing, 2001). Fisetin, a dietary flavonoid compound, has been found to exhibit cancer growth inhibition ability *via* alterations in the cell cycle and induce apoptosis. Fisetin, when reacting with LNCaP cells, causes PCa suppression *via* the G1-phase of cell cycle arrest and modulates the networking of CKI-cyclin-CDK (Lall et al., 2016). Luteolin, also a flavonoid compound, significantly inhibits the proliferation of PCa cells *via* induction of apoptosis in LNCaP cells (Roell and Baniahmad, 2011). Atraric acid and N-butyl-benzene sulfonamide are the two novel AR antagonists, extracted from an evergreen tree *P. africanum*, that have been used in combinational therapy for the treatment of PCa (Chiu and Lin, 2008).

**FIGURE 2:**
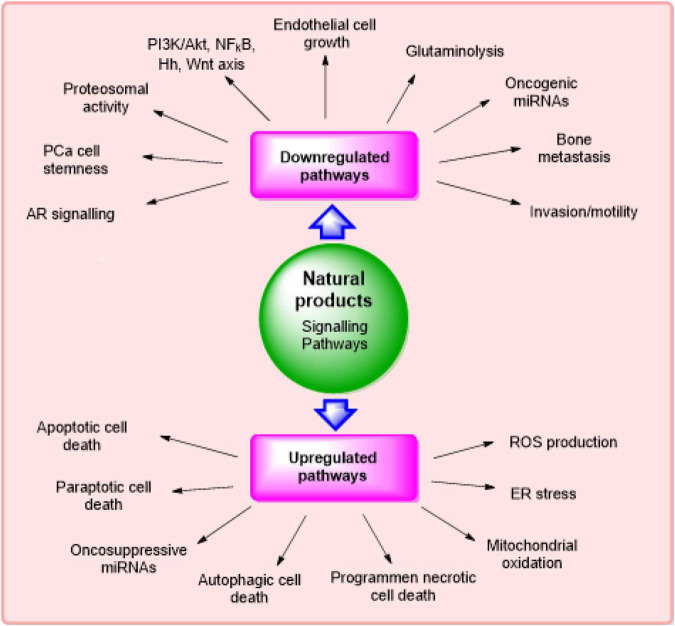
Major signaling pathways modulated *via* phytochemicals in management of castration-resistant prostate cancer.

According to literature survey, it has been found that several natural products and their derivatives/metabolites can selectively target the signaling pathways that are concerned with the expansion of cancer growth. The major signaling pathways modulated *via* phytochemicals in the management of CRPC (Fontana et al., 2020) are described in Figure 2.

Shamaladevi et al. (2013) showed that ericifolin (EF) or eugenol, extracted from Jamaican pepper berries, *Pimenta dioica* (allspice), inhibited the proliferation of prostate cancer cells *via* induction of apoptosis and formation of colonies. Meimetis et al. (2011) displayed in their research that niphatenones A and B, glycerol ether lipids extracted from *Niphates digitalis* (a marine sponge), inhibited the androgen-induced proliferation of LNCaP prostate cancer cells and acted as lead compounds for the production of newer medications used in the treatment of castration-resistant prostatic adenocarcinoma.

Sadar et al. (2008) showed that sintokamides A to E, extracted from a marine sponge *Dysidea* sp., inhibited the proliferation of prostate cancer cells *via* blockage of N-terminus trans-activation of ARs. Galardy et al. (2013) displayed that betulinic acid extracted from white-bark birch trees induced PCa-specific apoptosis *via* accumulation of poly-ubiquitinated proteins by inhibiting multiple deubiquitinases (DUBs).

Li et al. (2008) showed that the isoflavones present in soybeans inhibited the phosphorylation of Akt and FOXO3a and increased the expression of glycogen synthase kinase-3β (GSK-3β), which resulted in the down-regulation of AR and caused the induction of apoptosis and inhibition of the proliferation of PCa cells. Mori et al. (2006) and Malagarie-Cazenave et al. (2009) showed that capsaicin, a constituent of red peppers, inhibited the development and progression of androgen-independent p53 mutant PCa cells *via* PI3K and MAPK pathways in prostate LNCaP cells. It also inhibited the TNFα-stimulated degradation of IKBA in PC-3 cells, which has been associated with the inhibition of proteasome activity.

Cha et al. (2005) showed that emodin, extracted from the plant *Rheum palmatum*, inhibited the proliferation of PCa cells *via* down-regulation of androgen receptors from Hsp90 and intensification of its interactions with E3 ligase MDM2, thereby endorsing the proteasome-mediated deprivation of AR in LNCaP cells.

## Natural Product-Based Nanoparticles for Castration-Resistant Prostate Cancer

**FIGURE 3:**
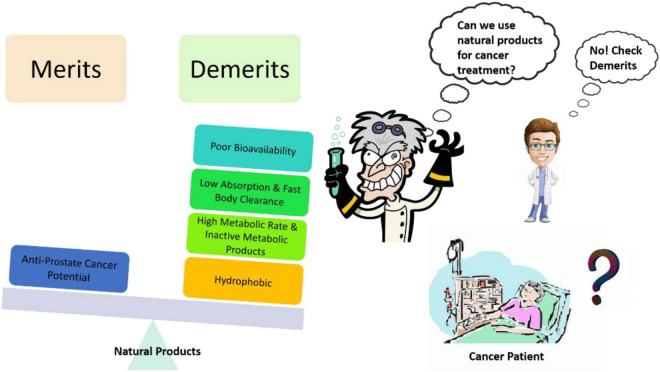
Common problems associated with natural products as an anti-prostate cancer agent and creating hurdles in their translational potential.

Site-targeted drug delivery is important while planning for chemotherapy as it will enhance the effectiveness of drugs and reduce the possibility of adverse reactions. Nanotechnology-based strategies not only support the doctors and researchers to achieve the site-targeted drug delivery but it also provides a great opportunity to explore new avenues that can impact the diagnosis, prevention, and therapy. While dealing with the potent natural products against CRPC, solubility, and bioavailability (Figure 3) are the unmet challenges, these issues could be addressed by nanoparticle formulations (Donaldson, 2004; Siddiqui et al., 2007, 2008; Syed et al., 2007; Khan et al., 2010; Bharali et al., 2011; Mukhtar, 2012; Siegel et al., 2012a,b; Wilson et al., 2012). Nanoparticulate therapies with unique characteristics, like a high surface area-to-volume ratio of the particle, and several biological properties provide solutions to the current issues in cancer treatment.

The objectives that we can achieve by involving nanotechnology are as follows: (a) we can obtain targeted drug delivery, (b) we can develop innovative diagnostic tools, (c) we can improve the pharmacokinetic drug profile, and (d) we can aid the transportation of diagnostic and therapeutic agents *via* biological barriers.

### Curcumin Nanoparticles in Castration-Resistant Prostate Cancer

Curcumin has various numbers of pharmacological activities such as antioxidant properties, anti-inflammatory action, capability to control the cell cycle, and stimulation of the process of apoptosis (Shishodia et al., 2007; Wilken et al., 2011). It also has the capacity to modulate autophagy and inhibit tumor angiogenesis and also metastasis in various cancers (Wang et al., 1997; Bar-Sela et al., 2010). The utilization of curcumin in clinical trials is limited due to its hydrophobic profile, instability, low pharmacokinetic profile, and poor water solubility, though it has many other beneficial pharmacological properties (Shoba et al., 2007). So, nanoparticles were developed for the effective delivery of curcumin to improve its efficacy in the treatment of cancers. Nanoparticles are potent to protect drugs from degrading, enhance drug stability, enhance controlled drug release, and also increase pharmacokinetic property and decrease toxicity profile of the drug (De Jong and Borm, 2008; Markman et al., 2013). Docetaxel is usually used in the mainstay treatment of CRPC. Over time, CRPC patients developed resistance against docetaxel, which might also be a reason behind the mortality of patients. Tanaudommongkon et al. (2020) used a few pharmaceutical excipients for the preparation of curcumin nanoparticles such as Miglyol 812 and a surfactant, d-alpha-tocopheryl polyethylene glycol succinate (TPEGS) 1000 (which is derived from vitamin E). The surfactant used in the preparation of nanoparticles has been approved by the Food and Drug Administration (FDA) as the safest excipients that can be employed in preparing many formulations. The inhibition of efflux by allosteric modulation of P-glycoprotein by TPEGS is also a valuable addition to selecting that particular surfactant (Collnot et al., 2010). Nanoparticles encapsulated with curcumin were prepared and characterized for zeta potential, particle size, drug loading, efficiency, differential scanning calorimetry analysis, stability study, and also *in vitro* studies.

### Quercetin and Its Nanoscale Delivery System

Quercetin shows certain undesirable features that may lead to its poor systemic availability. It has poor bioavailability and water solubility (0.00215 g/l at 25°C to 0.665 g/l at 140°C), and it is quickly metabolized by the body, which can limit its effectiveness as an agent for disease prevention (Ceña et al., 2012). The encapsulation of quercetin with biodegradable nanoparticles and biocompatibility may delay or avert its metabolism in the body and permit for retention of the long-term effect of quercetin in the blood and other tissues. Applying nanotechnology-based quercetin formulations can overcome the undesirable features to its delivery (Srinivas et al., 2010).

**FIGURE 4:**
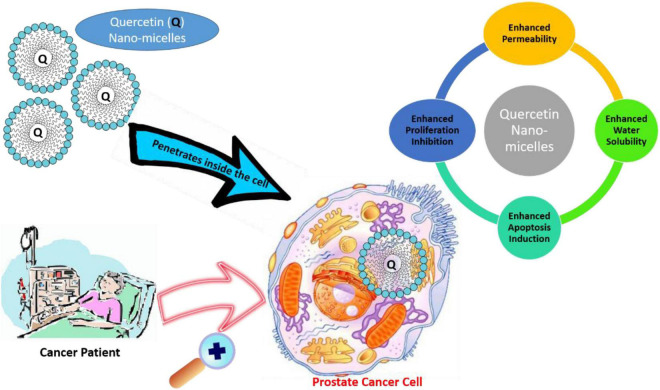
Application of quercetin nanomicelles in providing targeted delivery in prostate cancer therapy. These nanomicelles promote the penetration of quercetin into cancer cells, leading to an increase in bioavailability and subsequent enhancement.

To deal with the poor bioavailability and hydrophobicity of quercetin in CRPC, Zhao et al. (2016) conducted *in vivo* and *in vitro* studies by encapsulating quercetin in nanomicelles. An encapsulation of 1 mg/ml potentially improves the water solubility of quercetin 450-fold. The *in vitro* results showed that the IC_50_ for micellar quercetin formulation was 20 μM, compared to 200 μM of free quercetin. Therefore, the nano-based preparation capably inhibited proliferation and apoptosis in human androgen PCa cell lines. In addition to this, quercetin-loaded micelles *in vivo* showed higher antitumor efficiency, and proliferation rate decreased by 52.03% in comparison to the control group in the PC-3 xenograft mouse model, likely due to improved accumulation of micellar quercetin at the tumor site through enhanced permeability and retention effects (Zhao et al., 2016). The nanomicelle-based drug delivery system forms a promising and successful pharmaceutical treatment approach for PCa (Figure 4).

### Epigallocatechin Gallate and Its Nanoparticles

As already explained in the *Natural Therapeutic Products for CRPC Treatment and Management* section, EGCG is having the potential to manage CRPC, but bioavailability is an important concern. Rocha et al. (2011) had encapsulated EGCG in a polysaccharide matrix of gum arabic and maltodextrin and studied its anti-PCa potential in Du145 prostate cancer cells. They found that encapsulated EGCG not only retained the anti-prostate cancer activity by reducing cell viability and apoptosis induction but also had enhanced effects when compared with free EGCG (Rocha et al., 2011). Khan et al. (2014) had prepared the oral formulation of chitosan nanoparticles, which were encapsulated with epigallocatechin-3-gallate, and these chitosan-EGCG nanoparticles of < 200-nm diameter were then assessed for antitumor potential in athymic nude mice (subcutaneously implanted with 22 Rν1 tumor xenografts). Results indicated that the chitosan-EGCG nanoparticles were the better anti-PCa agent, as compared to EGCG alone or control groups, *via* modulation of multiple pathways. In the intestinal fluid, the release of EGCG was faster, thus addressing the bioavailability issue (Khan et al., 2014).

Combination therapies are quite trendy when dealing with chronic diseases because of synergy effects. Chen et al. (2020) had encapsulated EGCG with low doses of docetaxel (DTX) in the nanoparticles based on TPGS-conjugated hyaluronic acid and fucoidan. Out of multiple combinations, they found that an EGCG/DTX ratio 2.00:0.20 mg/ml had better and narrower distribution and significant percentage drug loading efficiencies. In the *in vitro* studies, they found internalization of these nanoparticles into prostate cancer cells, thus making the combination more target specific. Furthermore, *in vivo* studies indicated an increase in M30 protein expression with attenuated tumor growth, without affecting organs (Chen et al., 2020).

### Embelin and Its Nanoparticles

Lu et al. (2013) had prepared stable micelles with polyethylene glycol 5000 and embelin and then encapsulated paclitaxel in it to make a combination therapy. The nanomicelles were in the range of 20–30 nm. *In vitro* studies suggested an efficient uptake of these nanomicelles in the tumor site along with the slow release of paclitaxel. Furthermore, these nanomicelles exhibited much better cytotoxicity than individual drugs when tested in many tumor cell lines including DU145 and PC-3. *In vivo* studies suggested that these nanomicelles exhibited a significant safety profile with selective uptake in the tumor site, minimal movement in sensitive organs like the liver and spleen, and a maximum tolerated dose of 100–120 mg of paclitaxel/kg in mice. Overall, this combination of embelin and paclitaxel nanomicelles offered superior anti-prostate cancer activity as compared to paclitaxel in mouse models of prostate cancer (Lu et al., 2013). Danquah et al. (2009) had prepared the nanomicelles of polyethylene glycol-b-polylactic acid and encapsulated embelin and bicalutamide in them. They performed *in vitro* experiments on prostate cancer cell lines, namely, LNCaP and C4-2, while the *in vivo* studies were performed on BALB/C nude mice with LNCaP xenografts. This combination of embelin and bicalutamide had shown synergism in C4-2 with slight antagonism in the case of the LNCaP cell line. Furthermore, the aqueous solubility of this combination was increased by more than 60-fold in the nanomicelle form. Results from the *in vivo* studies also suggested that the combination in nanomicelle form was much better in regressing these hormone-refractory tumors (Danquah et al., 2009).

### Betulinic Acid and Its Nanoparticles

Saneja et al. (2017) had prepared nanoparticles of betulinic acid with polylactide-co-glycolide-monomethoxy polyethylene glycol and then studied it against the PANC-1 tumor cell line. The nanoparticles were approximately 147 nm in size and spherical. These betulinic acid nanoparticles were found to have an enhanced half-life by approximately 7.21-fold. Furthermore, the cytotoxic effect was also enhanced in the nanoparticle form, and it was seen because of improvement in apoptotic effect, mitochondrial membrane potential loss, high ROS level, and cell cycle arrest (Saneja et al., 2017).

Though nanoparticles of other natural anti-prostate cancer (anti-PCa) agents were also prepared, those nanoparticles were not discussed here because, to the best of our knowledge, the activity studied for the nanoparticles in those articles was not anti-PCa, for example, fisetin nanoparticles (Kadari et al., 2017), luteolin (Majumdar et al., 2014; Li et al., 2020), eugenol (Majeed et al., 2014), capsaicin (Elkholi et al., 2014; Peng et al., 2014; Lv et al., 2017; Hazem et al., 2021; Kunjiappan et al., 2021), and emodin (Sevilla et al., 2009; Wang et al., 2014; Wu et al., 2017; Jänicke et al., 2021). So, further research can be done to analyze the anti-prostate cancer potential for these nanoformulations. To the best of our knowledge, there are natural products like atraric acid, niphatenones A and B, sintokamides A to E, etc. for which nanoformulations are not yet designed.

## Clinical Studies for Natural Product-Based Castration-Resistant Prostate Cancer Management

Medicinal plants present themselves as a rich natural reservoir of diverse phytochemicals with untapped therapeutic potential (Cragg and Newman, 2013; Zulkipli et al., 2015). Reportedly, several phytochemicals were demonstrated to possess remarkable cytotoxic activities, making them potentially valuable as anti-cancer therapeutics (Orang-Ojong et al., 2013; Seca and Pinto, 2018). Of these, Taxol^®^ (paclitaxel), a plant alkaloid obtained from *T. brevifolia* is used in chemotherapy against various cancer types, including prostate cancer (Weaver and Bement, 2014; Zulkipli et al., 2015). Another plant-derived anti-cancer drug is Synribo^®^ (omacetaxine mepesuccinate), a plant alkaloid obtained from *Cephalotaxus* sp. (bark extracts), is used in the treatment of chronic myeloid leukemia (Seca and Pinto, 2018). These significant pharmacological developments in the phytochemical-based anti-cancer drugs encouraged further clinical trials for natural compounds as potential chemotherapeutic drugs (Butler et al., 2014). Quite dismally, only a few phytocompounds have been tested in clinical studies to evaluate their anti-CRPC potential. Furthermore, the tested phytochemicals failed to exhibit promising anti-cancer properties in these clinical trials. For example, cabazitaxel (Jevtana^®^), a semi-synthetic Taxol derivative, entered phase III of the CRPC clinical trial, but could extend the life expectancy in the inflicted patients by only 90 days (Paller and Antonarakis, 2011). Combination therapies employing synthetic drugs and phytochemicals were shown to be effective in some clinical trials for CRPC, albeit in initial phases only (phase I or phase II). For instance, in phase II clinical trial, combination therapy with curcumin (derived from *C. longa*) and docetaxel (plant alkaloid) demonstrated an elevated PSA response rate in the CRPC-inflicted patients (Mahammedi et al., 2016).

The clinical trials for natural products are discussed to guide the future studies that may use their nanoformulations. This unveils the fact that developing nanoparticle-based natural product therapeutics for CRPC management is still a long way off.

**FIGURE 5:**
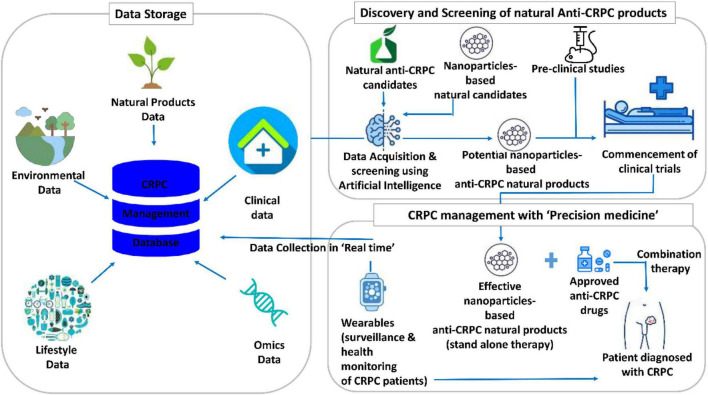
Translational informatics model for castration-resistant prostate cancer (CRPC) management using nanoparticles-based anti-CRPC natural products.

Figure 5 illustrates the translational informatics-based futuristic model for CRPC management using nanoparticle-based anti-CRPC natural products. For storing multilevel data, such as natural product data, omics data, lifestyle and environmental data, and clinical data, huge databases are needed. At present, the natural product databases store data on the putative candidates for several diseases. However, given the accumulating research data on natural product-based CRPC therapeutics and disease complexity, CRPC-specific natural product databases integrated with a systematic analysis are essential. These databases could be quite resourceful in providing the knowledge and references for artificial intelligence (AI) training. The developed AI systems are expected to be well-trained and perform crucial tasks. Specifically, these include the utilization of the AI system for screening natural products and/or their nanoformulations and recommendation of potential nanoparticle-based anti-CRPC natural products for preclinical studies and clinical trials. These studies, then, precisely reveal the effective nanoparticle-based anti-CRPC natural products, which can either be administered as a stand-alone or combination therapy (with the approved anti-CRPC drugs) to the CRPC patients. Another important task of AI is to perform surveillance and health monitoring of the patients diagnosed with CRPC. For this, the real-time physiological data of the patient are acquired using wearables and cloud platform and used as a reference. The integrated cloud platform-assisted daily analysis of the patient’s health provides suggestions on self-care for the CRPC patients with simultaneous reporting of their health status. This systemic model for CRPC care is quite promising, albeit several issues are to be addressed to fulfill it.

## Future Research Perspectives

The present study attempts to provide an overview of the intersection of nanomedicine and natural compounds in CRPC. It provides insights and guidance for future studies investigating the impact of natural product-based nanoparticles on CRPC in preclinical and clinical studies. Relevant future studies may benefit from a systematic review–meta-analysis methodology assessing both the then-available quantitative and qualitative evidence. Disruptive technology can become a game changer in future research.

Artificial intelligence models based on deep-machine learning can simulate, model and analyze natural compound-based nanoparticles, their bioavailability, and their interaction with therapeutic targets or other medicines (Miao et al., 2021; Shen et al., 2021). Prostate organoids can help simulate the tumor microenvironment and assess the effect of nanoformulations in a more realistic context, before moving toward animal and clinical studies. Such models have already been used to assess the effect of immunotherapeutic agents on PCa (Lakshmanan et al., 2021).

The translational research model should also be integrated with future studies. Given the therapeutic challenges of CRPC, patients can benefit from combination treatments including safe natural compound-based nanoparticles. In this case, the efficacy of these nanomedicines can be studied concerning particular genetic traits, biomarkers, or clinical features of the patients. The clinical outcomes can be compared with control groups, and their statistical significance can be improved by employing power analysis. Certainly, the use of a broader number of natural compounds requires regulatory support. It might be possible in advanced CRPC under compassionate authorization (Tsagkaris and Kalachanis, 2020). Nonetheless, such studies might undermine the efficacy of natural product nanoformulations due to the poor prognosis of the patients.

Finally, it is important to integrate social and health economic parameters in this research. Conducting appropriate research and integrating these medicines in an evidence-based and advanced technological context will make them more appealing to people who would object to their use in other circumstances (Chartterjee et al., 2005). The preparedness of researchers, clinicians, and patients to receive such medicines can be assessed through cross-sectional studies. These studies may adhere to a conventional questionnaire-based design, but they can also be based on social media and patients’ fora. Similar analyses have been conducted on Twitter polls about the acceptance of COVID-19 vaccines (Eibensteiner et al., 2021).

To the best of our knowledge, this article discusses all the major research developments in natural product nanoformulations for the management of CRPC. However, there are certain limitations, which need consideration. PubMed and Google Scholar were searched, and articles that were in English having DOI or PubMed numbers were preferred. Quite possibly, some related or crucial developments could have been missed out due to the exclusion of those articles that did not follow these criteria. Furthermore, this work does not discuss the nanoformulations of natural products that were not screened for anti-prostate cancer activity. Here, it is worth mentioning that the search of natural products against prostate cancer may not be exhaustive, but the nanoformulations for the enlisted natural products have been discussed in detail covering all crucial aspects, including translational research.

## Conclusion

PCa, a hormonally driven cancer, is associated with high morbidity and mortality toll among males worldwide. Castration resistance has been associated with more than 150 compounds, interacting with androgen receptors. The receptors, the binding compounds, and their biosynthetic pathways consist of therapeutic targets, which can be addressed by many natural compounds, including curcumin, quercetin, ericifolin, eugenol, niphatenones A and B glycerol ether lipids, sintokamides A to E, isoflavones present in soybeans, and emodin, extracted from the plant *R. palmatum*. Although these compounds manifest *in vitro* and *in vivo* anticancer activity, their potential is compromised in clinical settings. This could be credited to their poor pharmacodynamics and pharmacokinetic properties. Nanoparticle formulations can be used to modulate the biodistribution and target site accumulation of natural compounds, which, in turn, finely tunes the balance between their efficacy and toxicity. Undoubtedly, nanoformulations of natural products are comparatively costlier over the use of natural products alone. However, this is undermined by the associated advantages, such as the improved target specificity, increased activity with reduced side effects or adverse effects, bioavailability, slow release, and improved half-life offered by them. Moreover, the cost does not proportionately increase with the social and health economic burden. Curcumin nanoparticles can enhance its unfavorable pharmacological properties, namely, its hydrophobic profile, instability, low pharmacokinetic profile, and poor water solubility. The potential of nanoparticles encapsulated with curcumin in terms of particle size, drug loading, stability, and distribution has been manifested in the *in vitro* studies. The same applies to quercetin nanomicelle-based drug delivery systems in PCa. More studies are necessary to assess the potential of nanoformulations based on betulinic acid, capsaicin, sintokamide A, niphatenones A and B, and atraric acid. Simultaneously, established phytocompounds based anticancer drugs such as cabazitaxel (Jevtana^®^) can benefit from nanoparticle drug design in future clinical studies. Digital health applications can improve retrospective and prospective research to assess natural compound-based nanoparticles more comprehensively (Kletecka-Pulker et al., 2021). Research in the field can also be enriched by the compassionate administration of such nanomedicines in patients with advanced disease under designated regulatory frameworks. Overall, natural product-based nanomedicines have a major potential in CRPC, which needs to be verified through appropriate preclinical and clinical research.

## Author Contributions

All authors listed have made a substantial, direct and intellectual contribution to the work, and approved it for publication.

## Conflict of Interest

RS, SJ, and SS are honorary-based associated with the iGlobal Research and Publishing Foundation (iGRPF), New Delhi, India. The remaining authors declare that the research was conducted in the absence of any commercial or financial relationships that could be construed as a potential conflict of interest.

## Publisher’s Note

All claims expressed in this article are solely those of the authors and do not necessarily represent those of their affiliated organizations, or those of the publisher, the editors and the reviewers. Any product that may be evaluated in this article, or claim that may be made by its manufacturer, is not guaranteed or endorsed by the publisher.
